# Fractal dimension analysis: A new tool for analyzing colony-forming units

**DOI:** 10.1016/j.mex.2021.101228

**Published:** 2021-01-12

**Authors:** Gisele Alborghetti Nai, Cesar Alberto TALAvera Martelli, Denis Aloísio Lopes Medina, Mayla Silva Cayres de Oliveira, Isadora Delfino Caldeira, Bruno Carvalho Henriques, Maria Júlia Schadeck Portelinha, Lizziane Kretli Winkelstroter Eller, Mariângela Esther Alencar Marques

**Affiliations:** a Department of Pathology, Universidade do Oeste Paulista (UNOESTE), Rua José Bongiovani, 700, 19050-680 Presidente Prudente, SP, Brazil; b Department of Surgery, Universidade do Oeste Paulista (UNOESTE), Rua José Bongiovani, 700, 19050-680 Presidente Prudente, SP, Brazil; c Laboratory of Clinical Analysis, Universidade do Oeste Paulista (UNOESTE), Rua José Bongiovani, 700, 19050-680 Presidente Prudente, SP, Brazil; d Medical College, Universidade do Oeste Paulista (UNOESTE), Rua José Bongiovani, 700, 19050-680 Presidente Prudente, SP, Brazil; e Department of Microbiology, Graduate Program in Health Sciences, Universidade do Oeste Paulista (UNOESTE), Rua José Bongiovani, 700, 19050-680 Presidente Prudente, SP, Brazil; f Department of Pathology, Botucatu Medical School, São Paulo State University (UNESP), Rubião Junior, s/n, 18618-970, Botucatu, SP, Brazil

**Keywords:** Prostheses and implants, Microbial colony count, Image processing, Computer-assisted

## Abstract

The gold standard for quantifying bacteria both in routine diagnostics and in research is plating followed by count of colony-forming units (CFU). But, manual CFU counting on plates is time-consuming and subjective. We evaluated fractal dimension as a new methodology for evaluating CFU. Twenty fragments of expanded polytetrafluoroethylene (ePTFE) synthetic vascular prosthesis and 20 silicone prostheses were embedded in bacterial suspensions and incubated. The prostheses were then sown in solid culture medium and incubated for 48 h. Petri dishes were photographed and analyzed by fractal dimension. There was correlation between the number of CFU in manual counting and the fractal dimension analysis (*p* = 0.0001). We demonstrated that fractal dimension is a useful method for microbiological analyses in researches. It makes CFU analysis easier and faster and can be used regardless of the culture medium.

• Petri dishes with different bacterial colonies were photographed with a digital camera under natural light.
• The images were binarized and analyzed with ImageJ^Ⓡ^'s “fractal dimension” tool.
• Fractal dimension analysis showed to be a good tool for evaluating the amount of colony-forming unit.

## Graphical abstract

**Figure:**
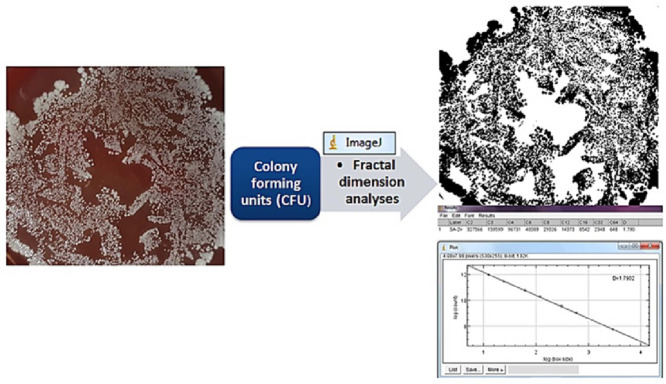

**Specifications Table**

**Table:** 

*Subject Area*	Microbiology
*More specific subject area*	Bacteriology
*Method name*	Colony-forming units counting (CFU)
*Name and reference of original method*	N. Stolze, C. Bader, C. Henning, J. Mastin, A.E. Holmes, A.L. Sutlief, Automated image analysis with ImageJ of yeast colony forming units from cannabis flowers, J Microbiol Methods. 164 (2019) 105,681. doi: 10.1016/j.mimet.2019.105681.
*Resource availability*	Example image files provided as supplementary material

## Method details

### Bacterial culture and counting procedure

*Bacterial strains and culture condition*

The bacterial strains (Microbiologics, Inc., St. Cloud, Minnesota, USA) used were *Escherichia coli* ATCC^Ⓡ^ 25,922™, *Proteus mirabilis* ATCC^Ⓡ^ 25,933™, *Staphylococcus aureus* subspecies *aureus* ATCC^Ⓡ^ 25,923™, *Staphylococcus epidermidis* ATCC^Ⓡ^ 12,228™ and *Enterococcus faecalis* ATCC^Ⓡ^ 29,212™.

About 50µL of bacterial frozen stocks samples (10^8^ CFU/ml) were inoculated in BHI (brain heart infusion) and incubated at 37 °C for 24 h.

### Prostheses colonization

Twenty fragments of the Exxcel Soft expanded polytetrafluoroethylene (ePTFE) synthetic vascular prosthesis (Maquet Cardiovascular LLC, Wayne, NJ, USA) measuring 1 cm each and 20 silicone prostheses measuring 2 cm diameter (Model Forma Malhas Compressivas e Produtos Hospitalares Ltda. - EPP, São Caetano do Sul, Brazil) were used. Both prostheses were soaked in suspensions in 1 ml of TSB (Tryptic Soy Broth) with the microorganisms. The suspensions with the microorganisms were adjusted to the turbidity corresponding to McFarland scale tube 0.5 which represents 1,5 × 10^8^ CFU/ml . Later, the prostheses were incubated in sterile flasks in an oven at 37 °C for one week. One milliliter of TBS was added to each flask every 2 days during the incubation. The analysis was performed in quadruplicate for each bacterium and prostheses.

After incubation, the prostheses were seeded by rolling in 15 × 150 mm Petri dishes containing 40 ml of CLED (Electrolyte Deficient Cystine Lactose Agar) for evaluation of *P. mirabilis* and 40 ml of blood agar for evaluation of other bacteria. The plates were incubated at 37 °C for 48 h.

### Digital acquisition of image

After incubation, the Petri dishes were photographed with a digital camera (Cyber Shot W830 20.1MP, Sony, Japan) at a distance of 15 cm.

### Fractal dimension analysis

The images were analyzed with ImageJ^Ⓡ^ software (http://rsbweb.nih.gov/ij/) [1].

**Fig. 1:**
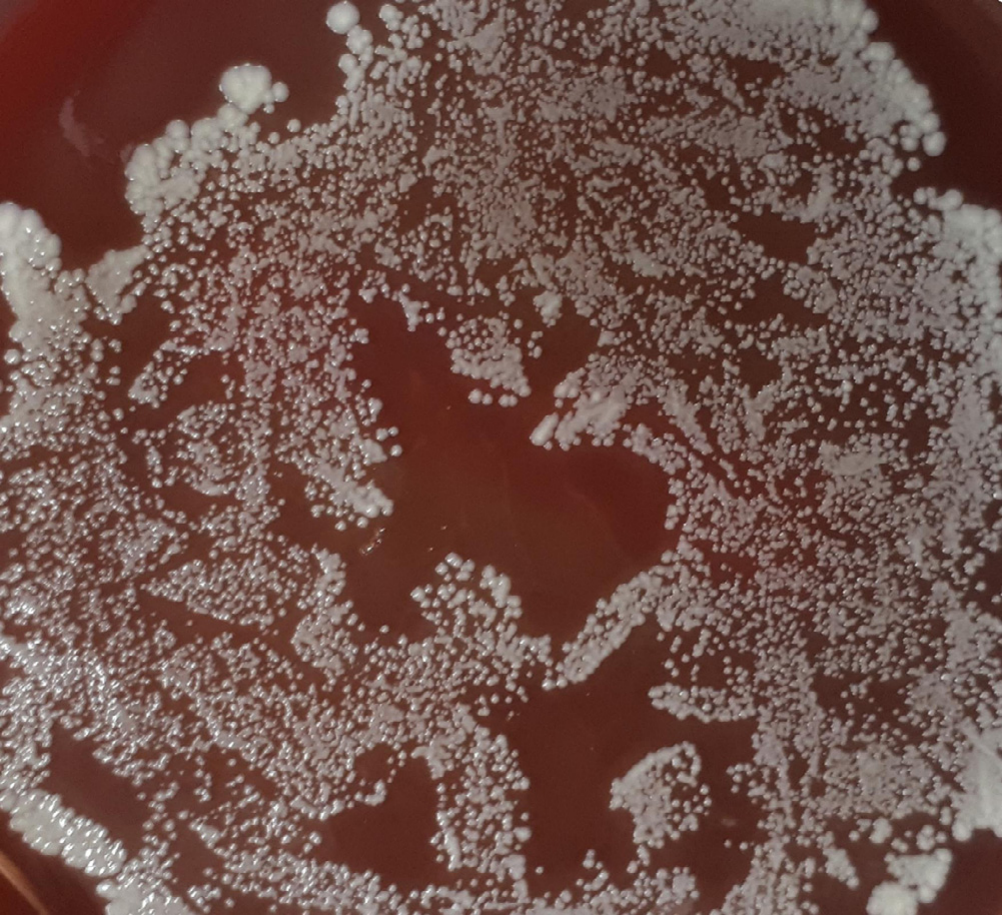
Original image of Petri dishes inoculated with ePTFE prostheses contaminated with *Staphylococcus aureus*. Culture medium: Blood agar.

**Fig. 2:**
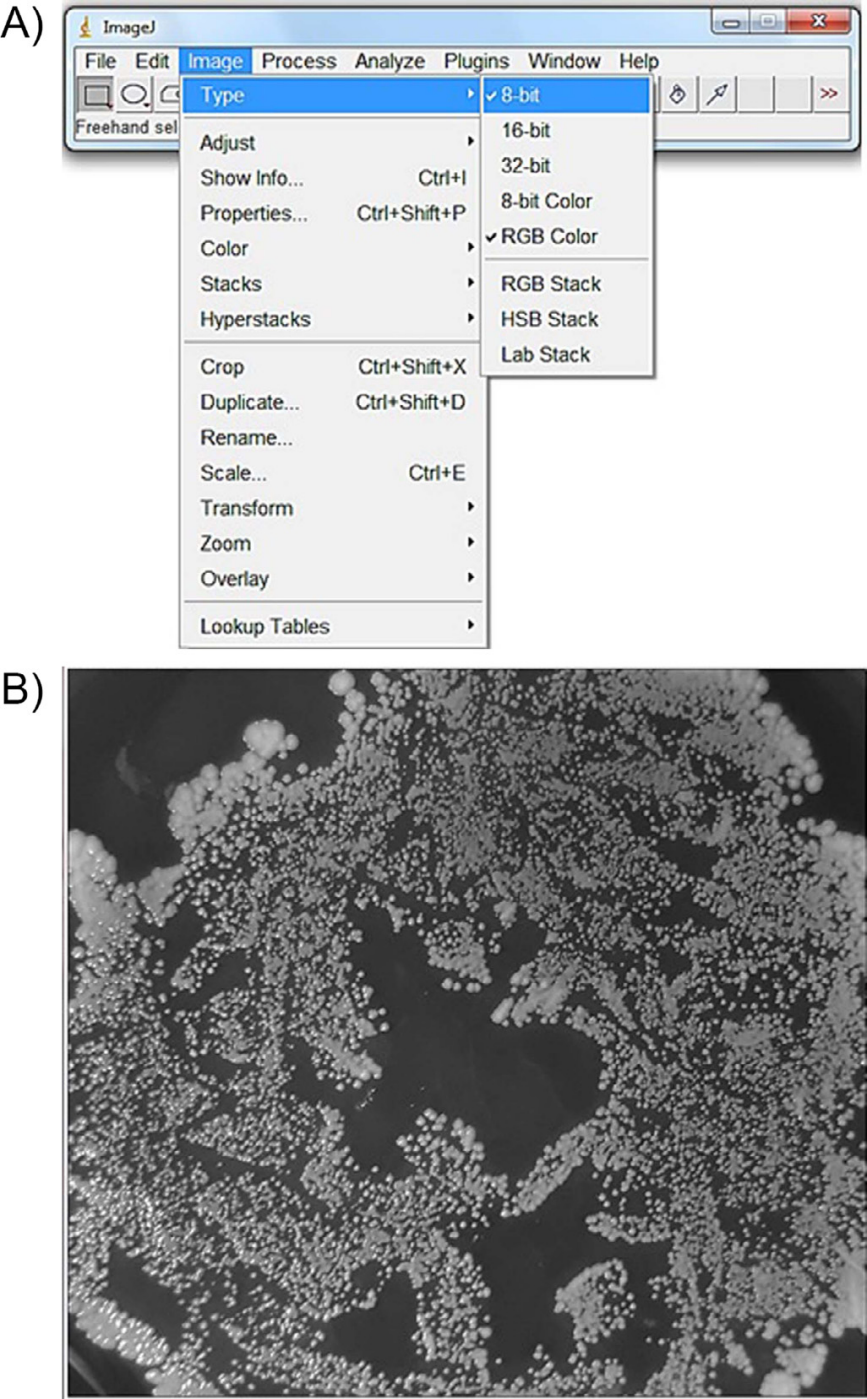
(a) “Image type” tool of ImageJ^Ⓡ^ and (b) original image transformed into 8-bit length.

**Fig. 3:**
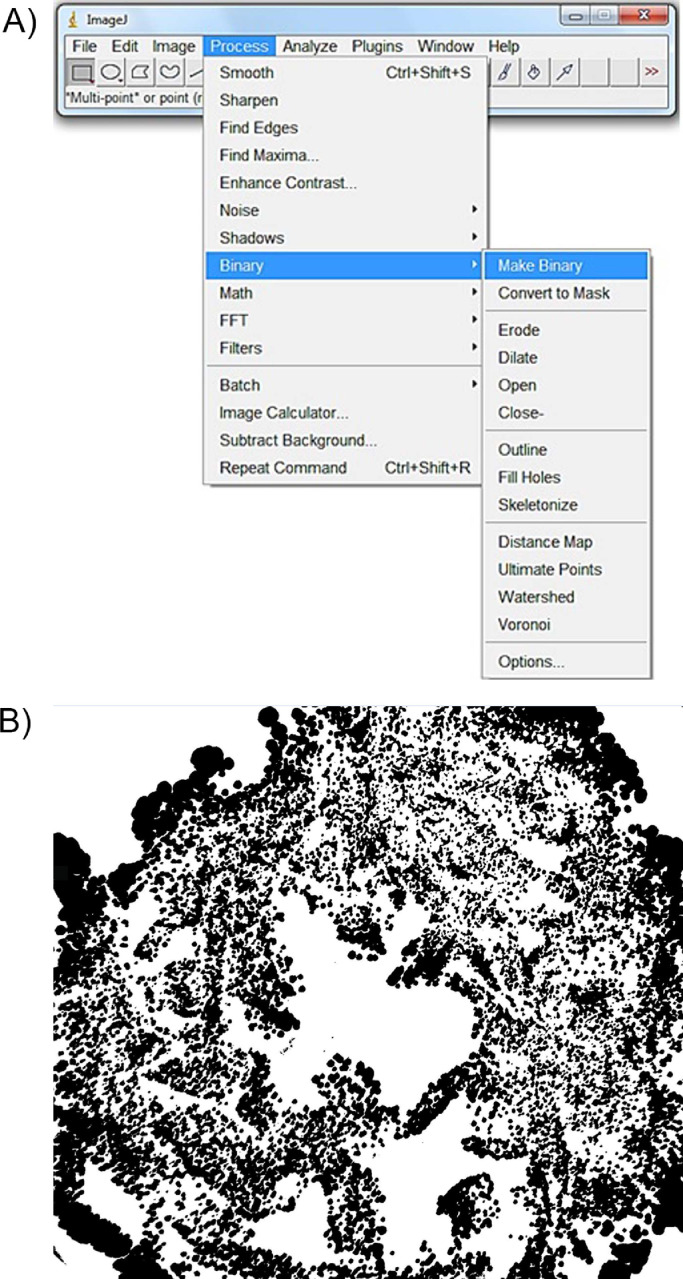
Binarization of the image: (a) the windows of ImageJ^Ⓡ^ for binarization and (b) the binarized image.

First, the original images (Fig. 1) were transformed into 8-bit length, using the “image type” tool (Fig. 2). Subsequently, they are binarized into black and white, using the “make binary” tool (Fig. 3), because fractal analysis measures the black area of the image.

**Fig. 4:**
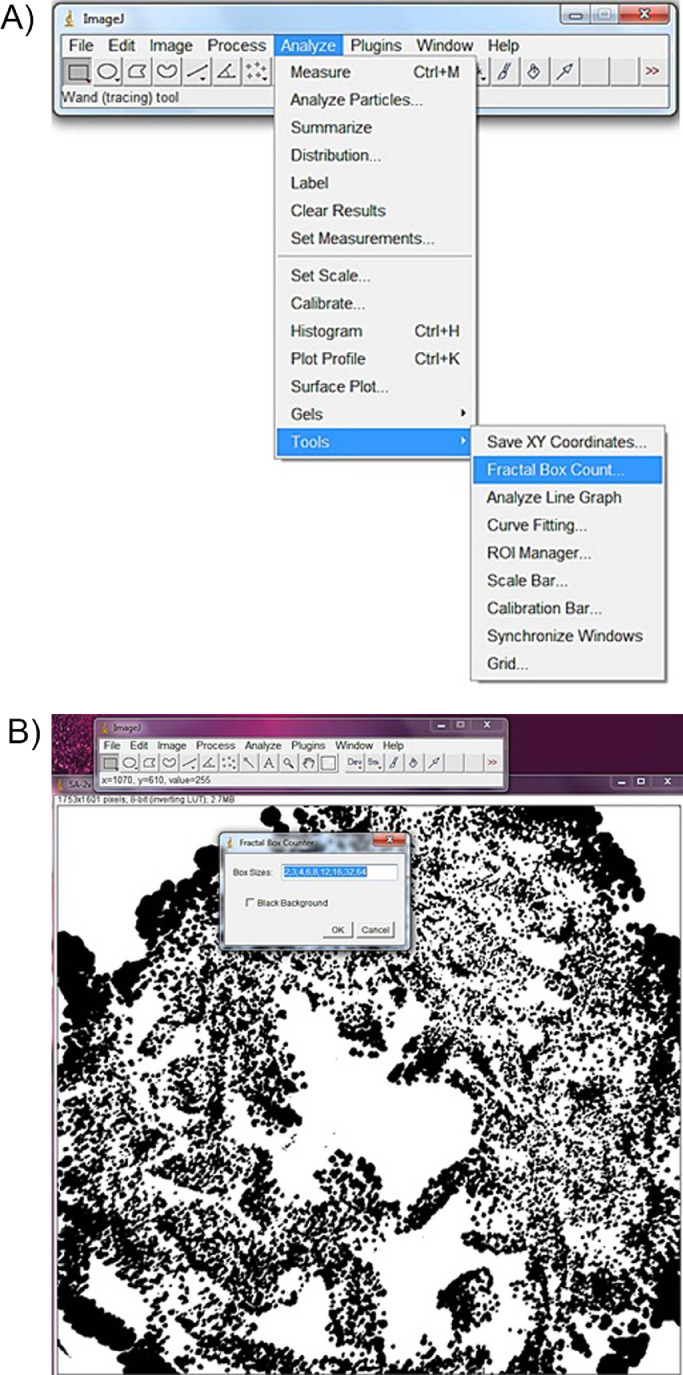
The windows of ImageJ^Ⓡ^ for “analyze” tool: (a) “Fractal box-counter” tool and (b) and the beginning of image analysis.

**Fig. 5:**
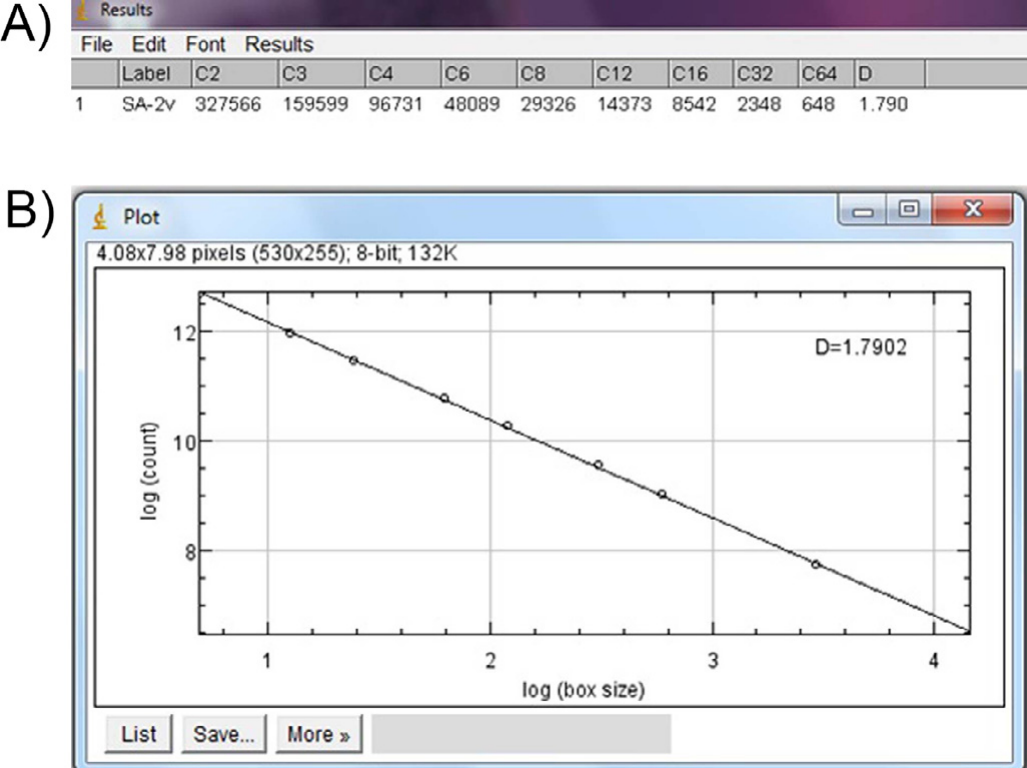
Results of fractal dimension analysis: (a) data spreadsheet and (b) the box-counting.

Fractal dimension analysis was performed by the box-counting method, using the “fractal box-counter” tool (Fig. 4). During the analysis, the program considers two dimensions, allowing the quantification of the pixel distribution in this space, not considering the texture of the image. The calculated fractal dimension is always between 0 and 2. The results are expressed in a spreadsheet and a boxplot (Fig. 5).

### Colony-forming units manual counting

Colony-forming units (CFU) were counted manually by the naked eye in the original image using the “multi-point” tool of ImageJ^Ⓡ^ software.

The CFU count ranged from 0 to 2893 on Petri dishes seeded with silicone prostheses and ranged from 0 to 3038 on Petri dishes seeded with ePTFE prostheses.

### Comparison between the CFU manual counting and the fractal dimension analysis

To check the appropriateness of using the fractal dimension to estimate the CFU, Pearson's correlation coefficient was used. Differences were considered statistically significant when *p* <0.05. The tests were performed with SPSS v. 23.0.

**Fig. 6:**
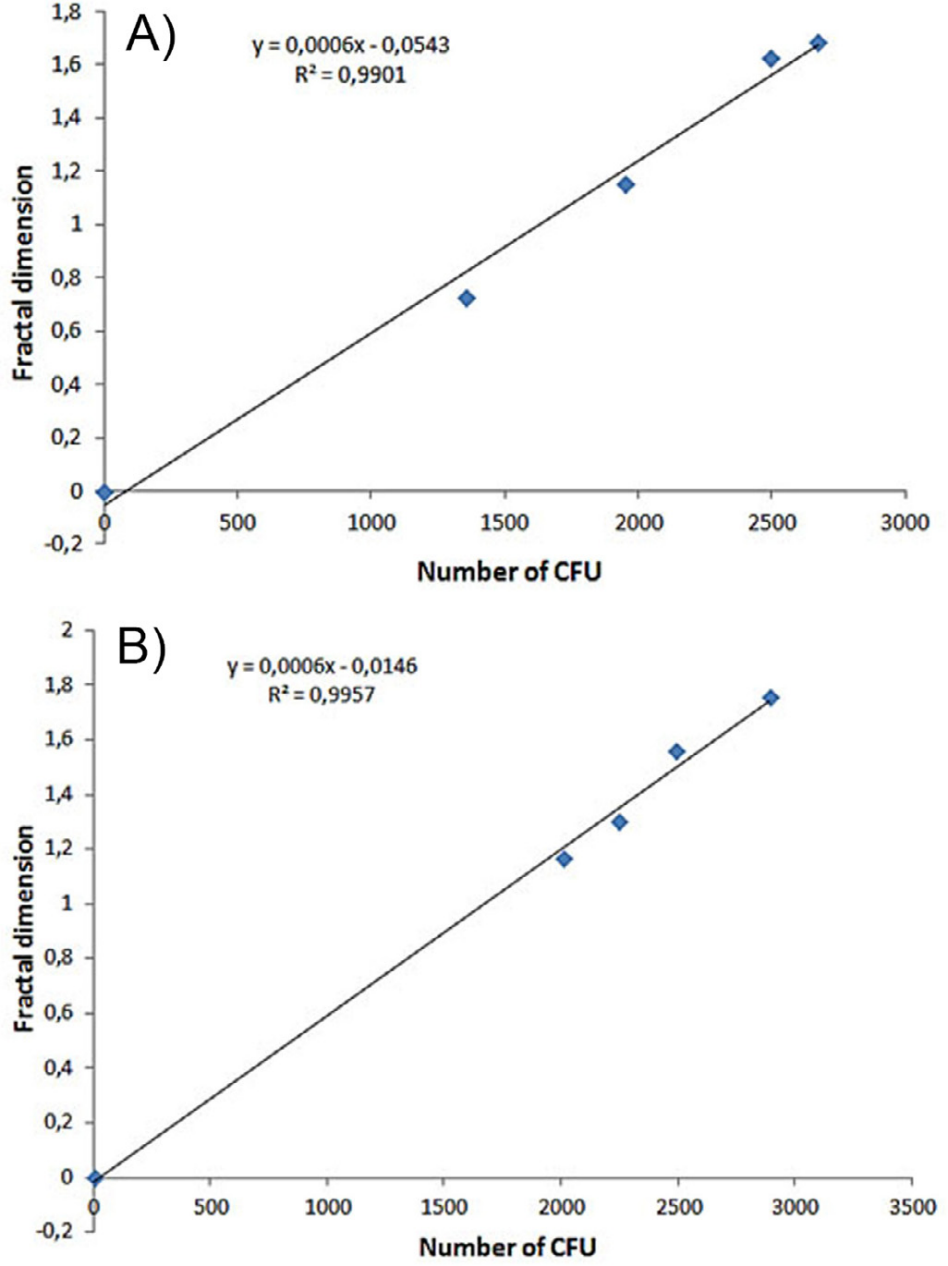
Correlation between the mean number of colony-forming units (CFU) counted manually and the analysis by fractal dimension for all bacteria evaluated: (a) of silicone prostheses (*p* = 0.0001) and (b) of ePTFE prostheses (*p* = 0.0001).

There was correlation between the number of CFU and the fractal dimension analysis of silicone prostheses (*r* = 0.995, *p* = 0.0001) (Fig. 6A) and ePTFE prostheses (*r* = 0.998, *p* = 0.0001) (Fig. 6B) for all the evaluated bacteria, regardless of their morphological characteristics.

## Additional information

### Background

The gold standard for quantifying bacteria both in routine diagnostics and in research is plating in a culture medium, incubation and later, the counting of colony-forming units (CFU) [2]. In addition to being a laborious method, manual CFU counting on plates is time-consuming and subjective [2,3]. The quantification of bacteria is very important for the characterization of both pathogen-host interactions and pathogenic factors involved [2].

Establishing a standardized procedure allows simplifying the process, making it easier and faster, in addition to decreasing the variability between analyzers [3], and it is necessary to obtain high performance analyzes [2].

Some high-throughput methods have been suggested for CFU counting, such as fluorescent labeling, genome probing microarrays, quantitative PCR, but these methods require specific equipment and a very detailed protocol with a lot of inputs [4]. Some artificial intelligence tools, more specifically machine learning, have been proposed to improve the performance of the CFU count, such as the Convolutional Neural Network proposed by Ferrari et al. [5]. However, this method is not suitable for high bacterial loads [4]. Thus, other machine learning methods that can evaluate a large number of colonies were also carried out, such as Transfer Learning that was first designed to count objects in a crowded scene [4]. But even machine learning requires complex data analysis.

The term fractal dimension was coined by the mathematician Mandelbrot in 1975, who stated that any set can have a fractal dimension [6]. The fractal dimension represents the degree of occupation of a geometric figure in space, i.e., it indicates the irregularity of the analyzed figure [6].

In medicine, fractal dimension analysis has been used in cardiovascular analysis [7], neurosciences [8] and pulmonary carcinoma analysis [9]. Fractal analysis for pathogen assessment was used in some studies previously [10], [11], [12], [13], but ours is the first to use the tool available by the software ImageJ^Ⓡ^.

The ImageJ^Ⓡ^ software is free and easy to use, which makes the analysis proposed in our study widely usable. Other studies propose software analyzes, but these require the use of more sophisticated equipment to capture the image and a software that often have a high cost. We propose the use of tools that are easily available to all researchers, are easily performed and interpreted and that are inexpensive.

Fractal dimension analysis is able to identify a small number of colonies and even small colonies (see Supplementary Figs. 1 and 3). As the fractal dimension evaluates tissue irregularity, even a small number of colonies will show changes in its value. Also, the fractal dimension was able to identify bacterial colonies in different culture media (see Supplementary figures). Regardless, we used plates seeded with contaminated prostheses in our study; fractal dimension analysis can be used in any context of CFU counting, because the analyses are made of the CFU on the Petri dishes with agar.

For the proper execution of the method, some care must be taken. The Petri dishes must all be photographed from the same distance and the size of the analyzed area must also be the same for all plates. Ideally, the camera should be attached to a support so that the distance is always the same and there is no image shake. The plates must be photographed on a flat surface, at a 90° angle to the floor, under white light. The projection of shadow on the plates prevents proper binarization of the images, making fractal analysis unfeasible.

The study also evaluated five different species of microorganism (*E. coli* ATCC^Ⓡ^ 25,922™, *Proteus mirabilis* ATCC^Ⓡ^ 25,933™, *Staphylococcus aureus* subspecies *aureus* ATCC^Ⓡ^ 25,923™, *Staphylococcus epidermidis* ATCC^Ⓡ^ 12,228™ and *Enterococcus faecalis* ATCC^Ⓡ^ 29,212™) and tested in two different culture media: CLED and blood agar. This confirms the relevant application of the method in different conditions. However, further studies evaluating different numbers and sizes of CFU, using other culture media and even other microorganisms, such as fungi, may better show the applicability of the fractal dimension analysis in microbiology.

The fractal dimension analysis showed an excellent correlation with the CFU manual count, showing that this can be a good tool for this type of evaluation, particularly in researches. As well as, the tool provided by the ImageJ^Ⓡ^ software made this analysis easier to perform than the methods previously described.

## Author contribution

Each author presented relevant contribution to elaboration of the present manuscript as follows:

Gisele Alborghetti Nai: coordinated the study, writer of the manuscript, performed prostheses analyses, and interpreted the results.

Cesar Alberto Talavera Martelli: co-writer of the manuscript and performed prostheses analyses.

Denis Aloísio Lopes Medina: co-writer of the manuscript and performed prostheses analyses.

Mayla Silva Cayres de Oliveira: performed prostheses analyses, and contributed reagents, materials, analysis tools or data and interpreted the results.

Isadora Delfino Caldeira: performed prostheses analyses.

Bruno Carvalho Henriques: performed prostheses analyses.

Maria Júlia Schadeck Portelinha: performed prostheses analyses.

Lizziane Kretli Winkelstroter Eller: co-writer of the manuscript, interpreted the results and contributed reagents, materials, analysis tools or data.

Mariângela Esther Alencar Marques: coordinated the study, revised the manuscript.

## Declaration of Competing Interest

The authors declare that they have no known competing financial interests or personal relationships that could have appeared to influence the work reported in this paper.

## Appendix Supplementary materials

Image, application 1
