# Supplementary material for: Fractal dimension analysis: A new tool for analyzing colony-forming units
Source: MethodsX. 2021 Jan 12;8:101228. doi: 10.1016/j.mex.2021.101228 (PMC8374233; doi:10.1016/j.mex.2021.101228)
Supplement: Supplementary file 1 [file mmc1.docx]

**Supplementary material**


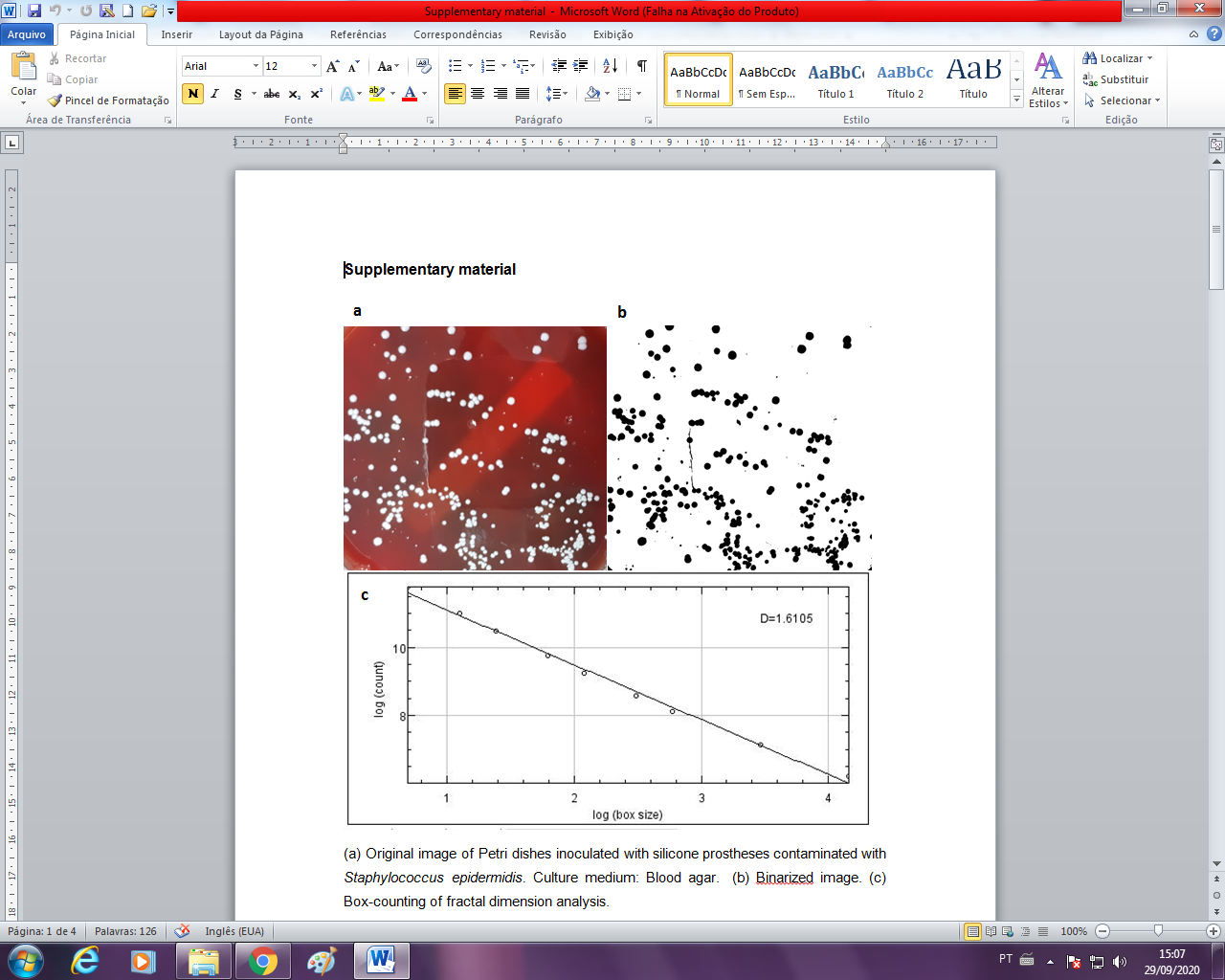


Supplementary figure 1. (a) Original image of Petri dishes inoculated with silicone prostheses contaminated with *Staphylococcus epidermidis.* Culture medium: Blood agar. (b) Binarized image. (c) Box-counting of fractal dimension analysis.


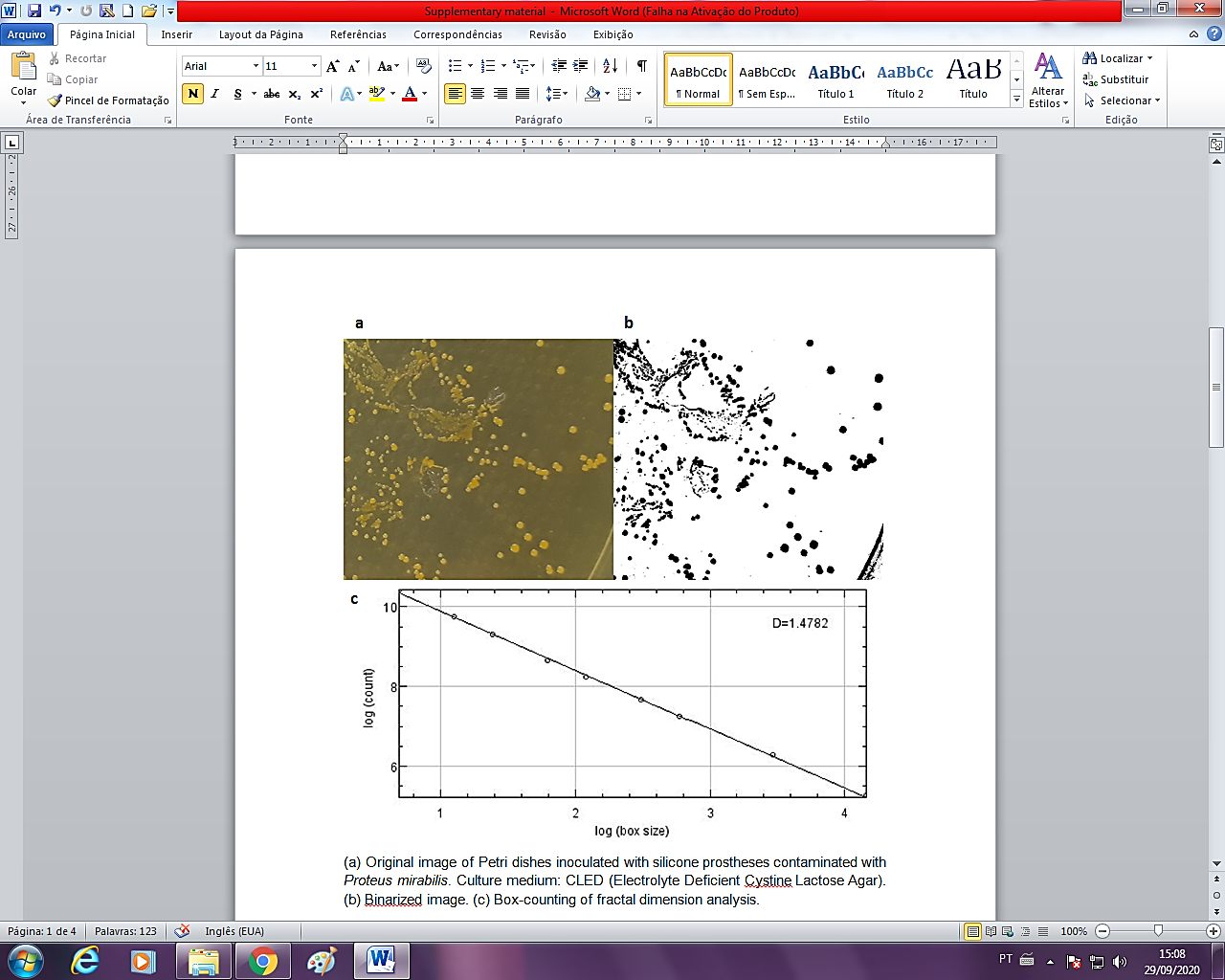


Supplementary figure 2. (a) Original image of Petri dishes inoculated with silicone prostheses contaminated with *Proteus mirabilis.* Culture medium: CLED (Electrolyte Deficient Cystine Lactose Agar). (b) Binarized image. (c) Box-counting of fractal dimension analysis.


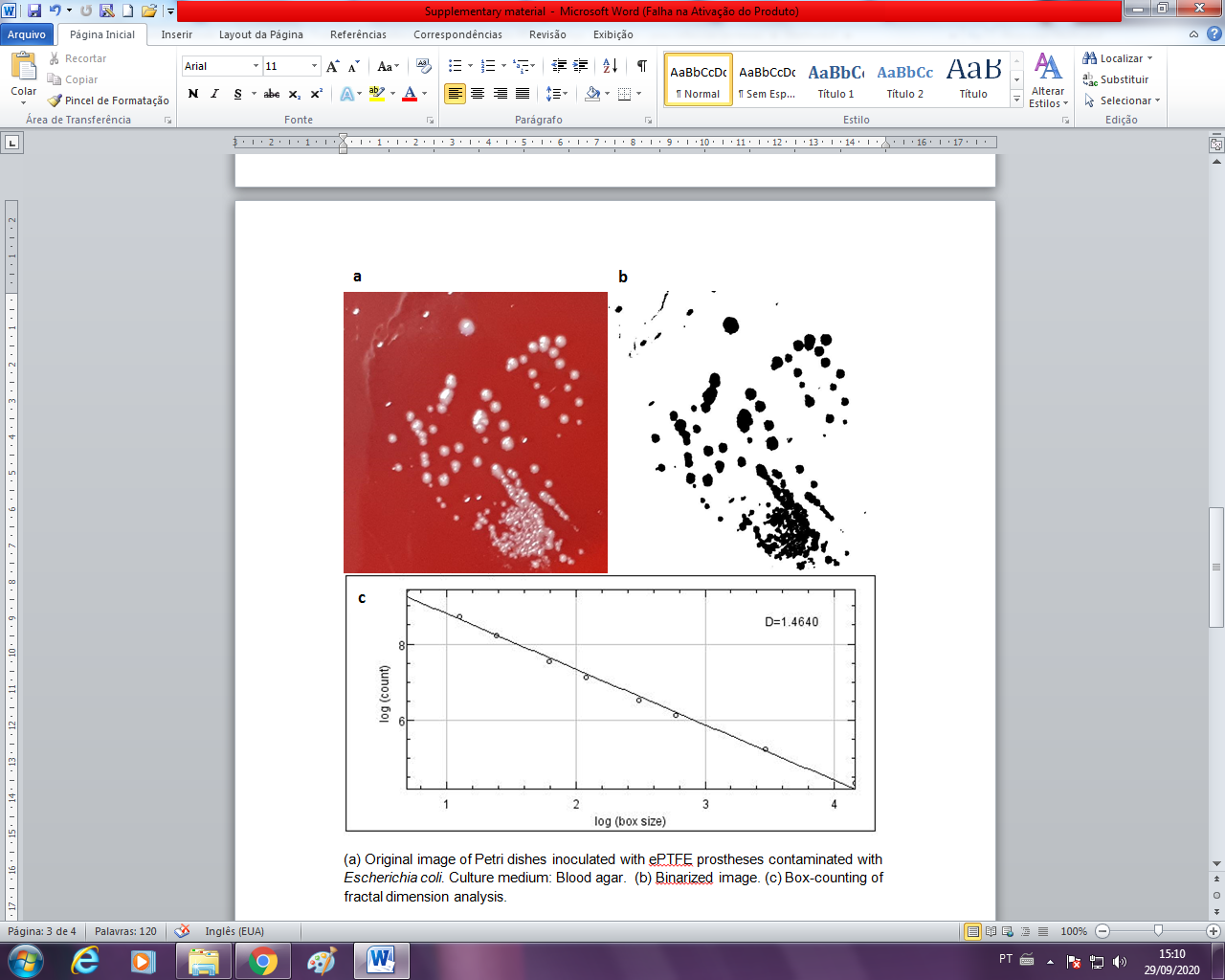


Supplementary figure 3. (a) Original image of Petri dishes inoculated with ePTFE prostheses contaminated with *Escherichia coli.* Culture medium: Blood agar. (b) Binarized image. (c) Box-counting of fractal dimension analysis.


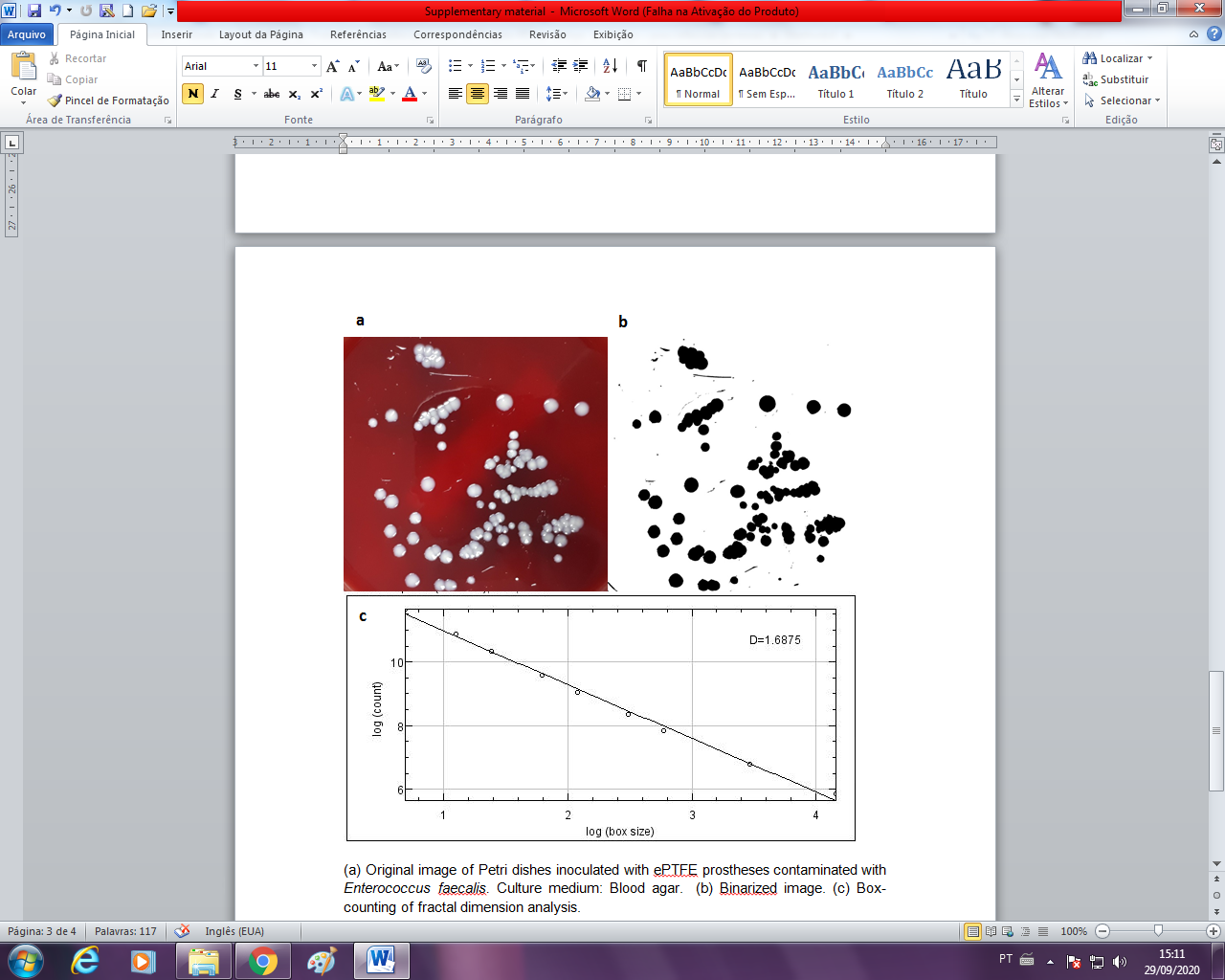


Supplementary figure 4. (a) Original image of Petri dishes inoculated with ePTFE prostheses contaminated with *Enterococcus faecalis.* Culture medium: Blood agar. (b) Binarized image. (c) Box-counting of fractal dimension analysis.
